# Supplementary figures and images for: The switch-like expression of heme-regulated kinase 1 mediates neuronal proteostasis following proteasome inhibition
Source: eLife. 2020 Apr 24;9:e52714. doi: 10.7554/eLife.52714 (PMC7224698; doi:10.7554/eLife.52714)

Figure 1- Source data

A

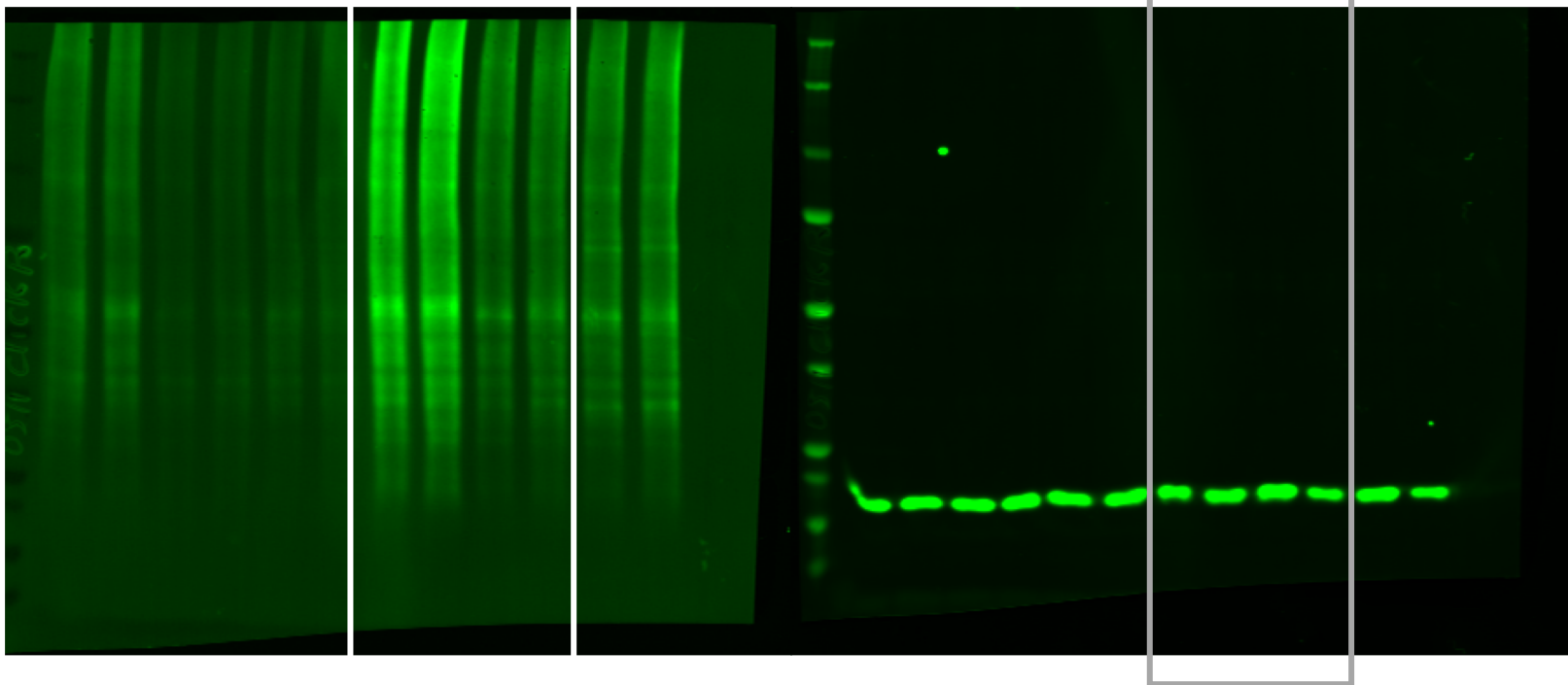

G

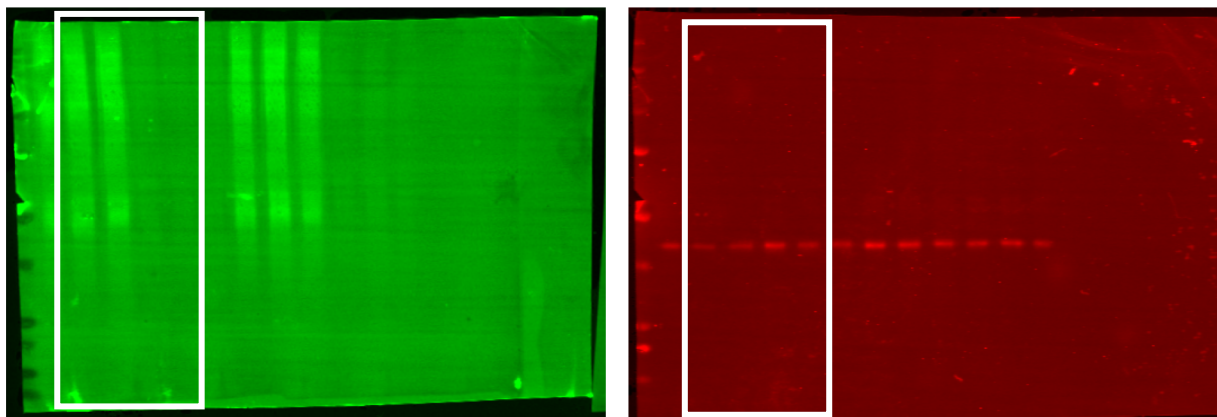

Supplement: Figure 1—source data 1. [file elife-52714-fig1-data1.pdf]

Figure 1- figure supplement 1- source data

Figure 1- figure supplement 1 C,D

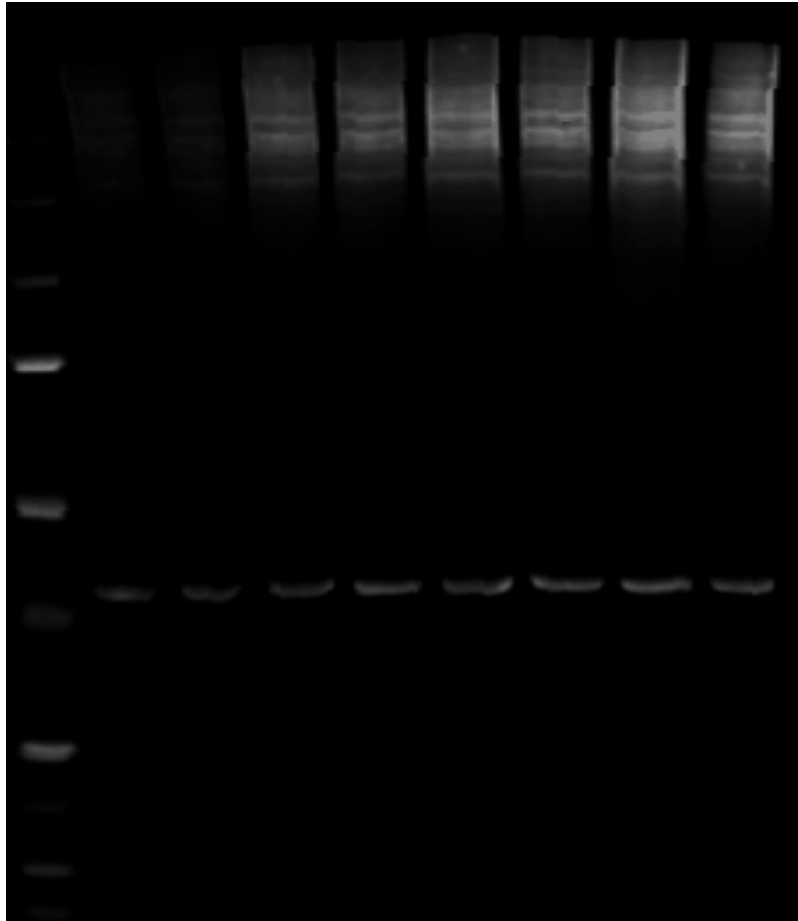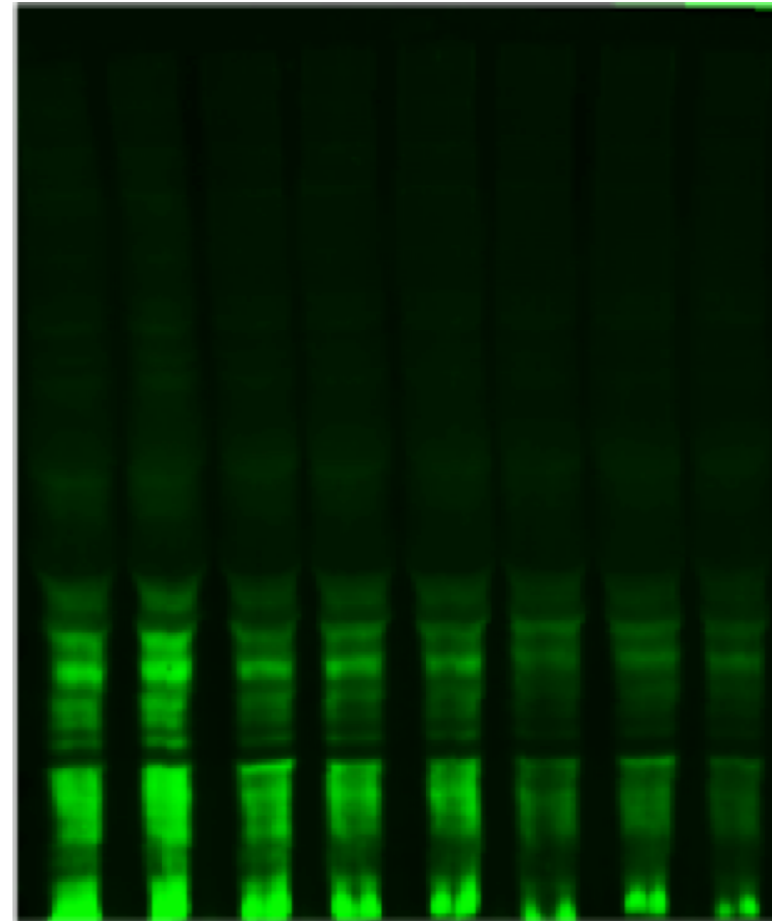

Supplement: Figure 1—figure supplement 1—source data 2. [file elife-52714-fig1-figsupp1-data2.pdf]

Figure 1- figure supplement 2- source data

Figure 1 figure supplement 2C

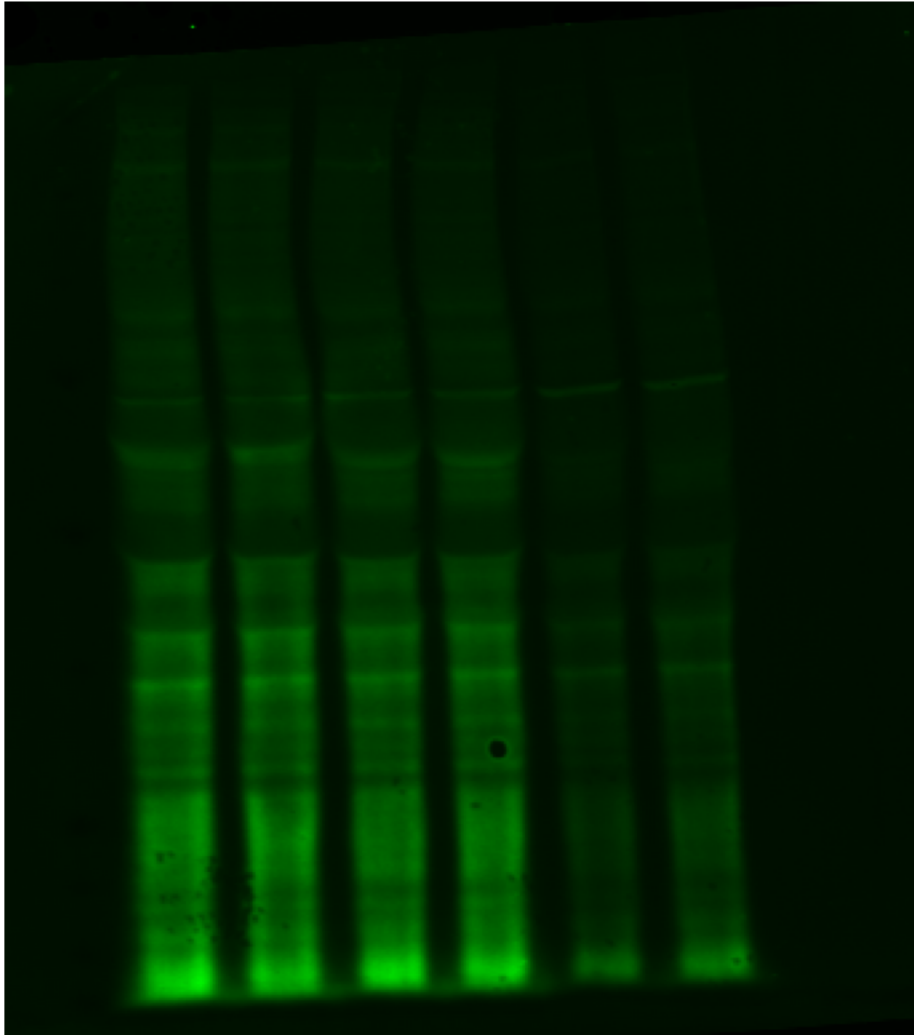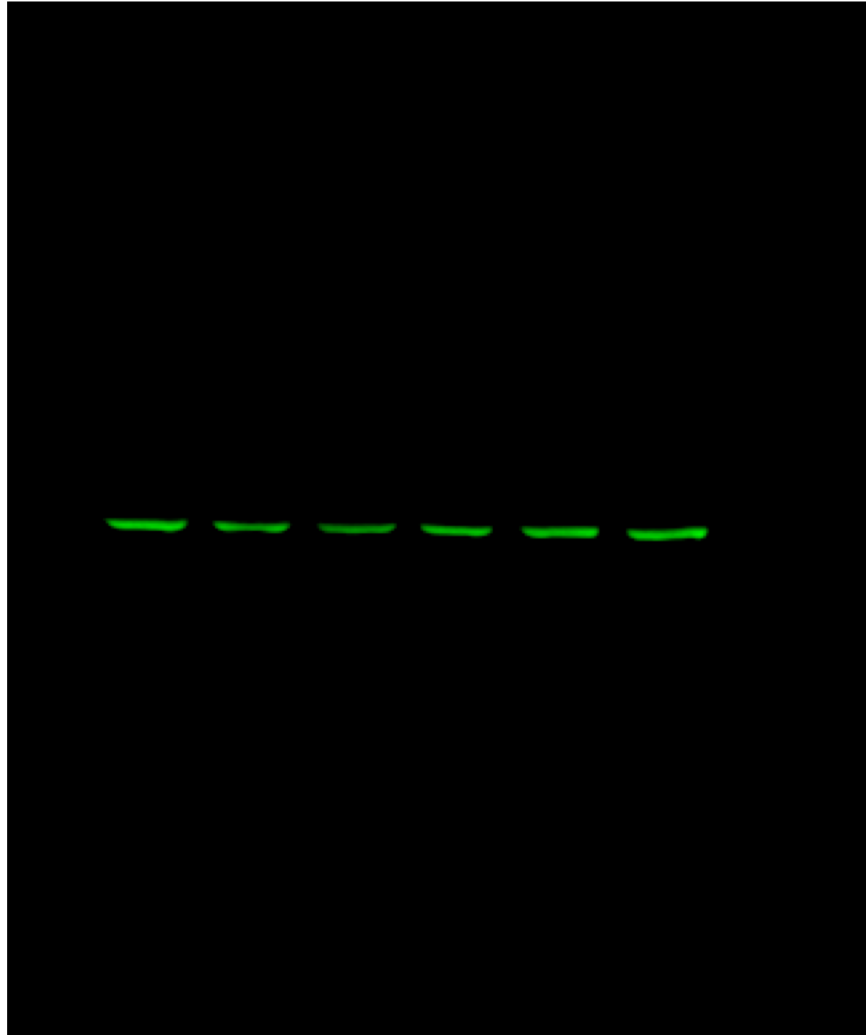

Supplement: Figure 1—figure supplement 2—source data 2. [file elife-52714-fig1-figsupp2-data2.pdf]

Figure 2- source data

**Figure 2A**

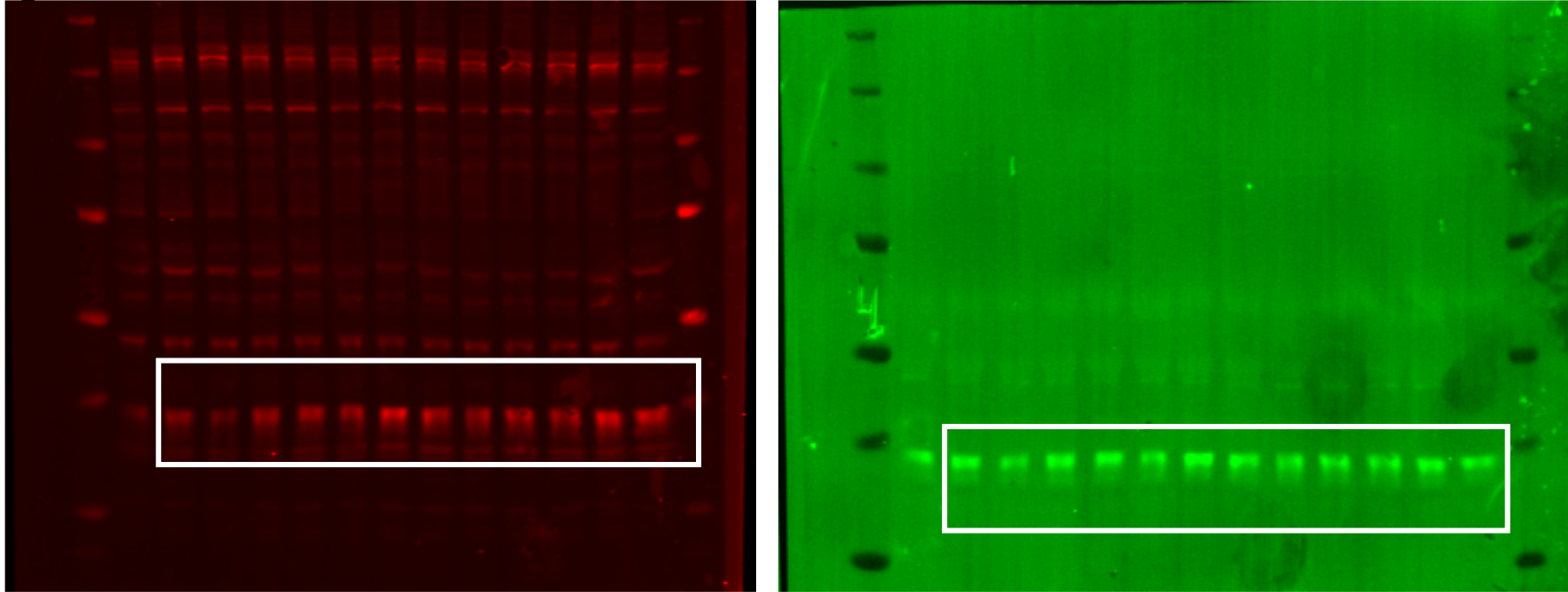

**Figure 2D**

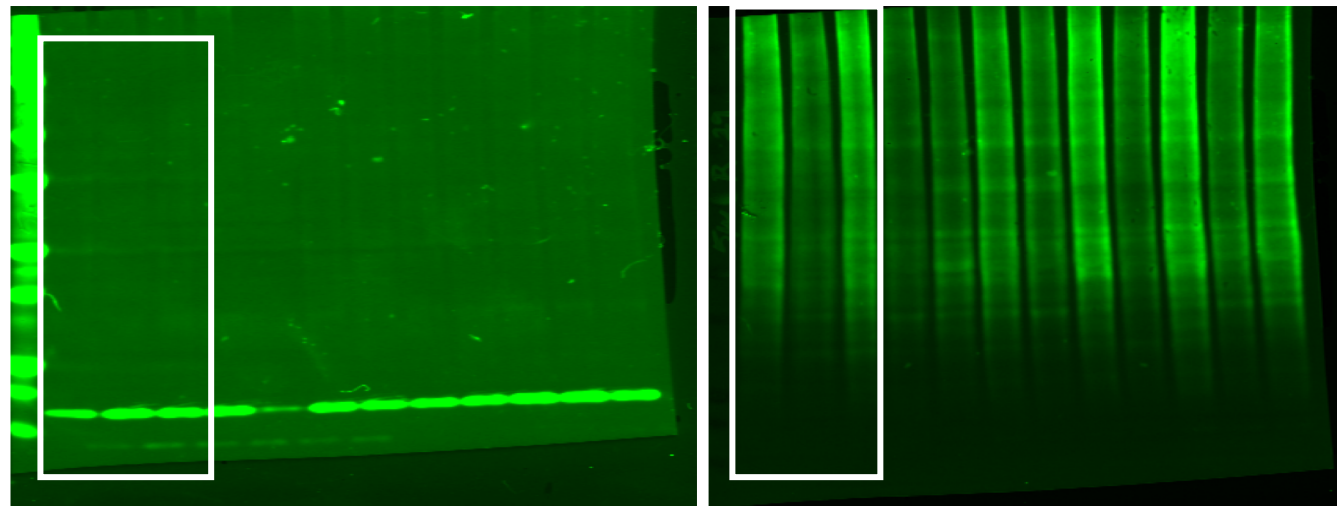

Supplement: Figure 2—source data 1. [file elife-52714-fig2-data1.pdf]

Figure 3B-Source data

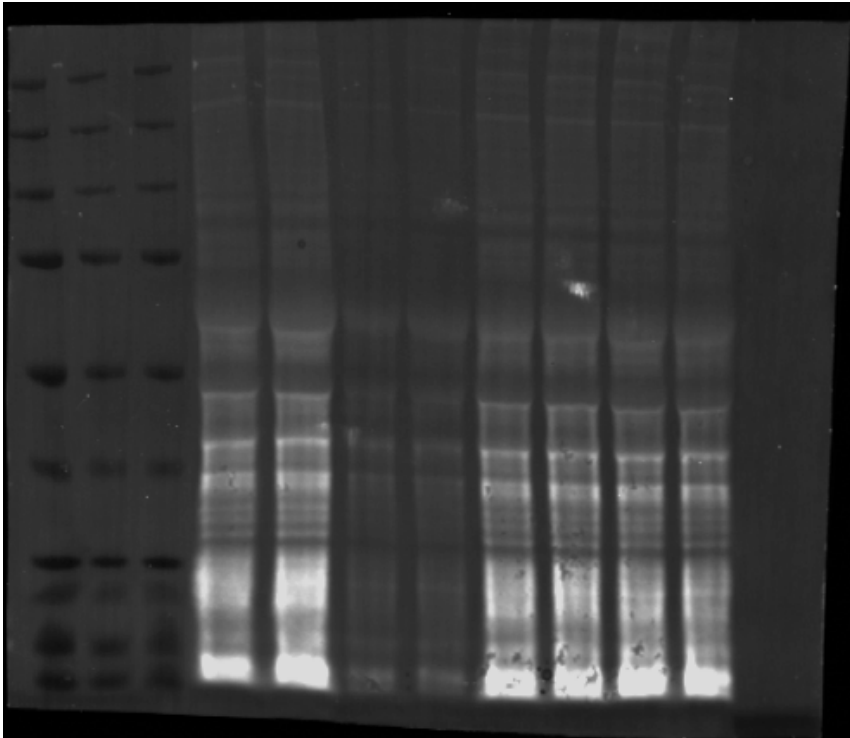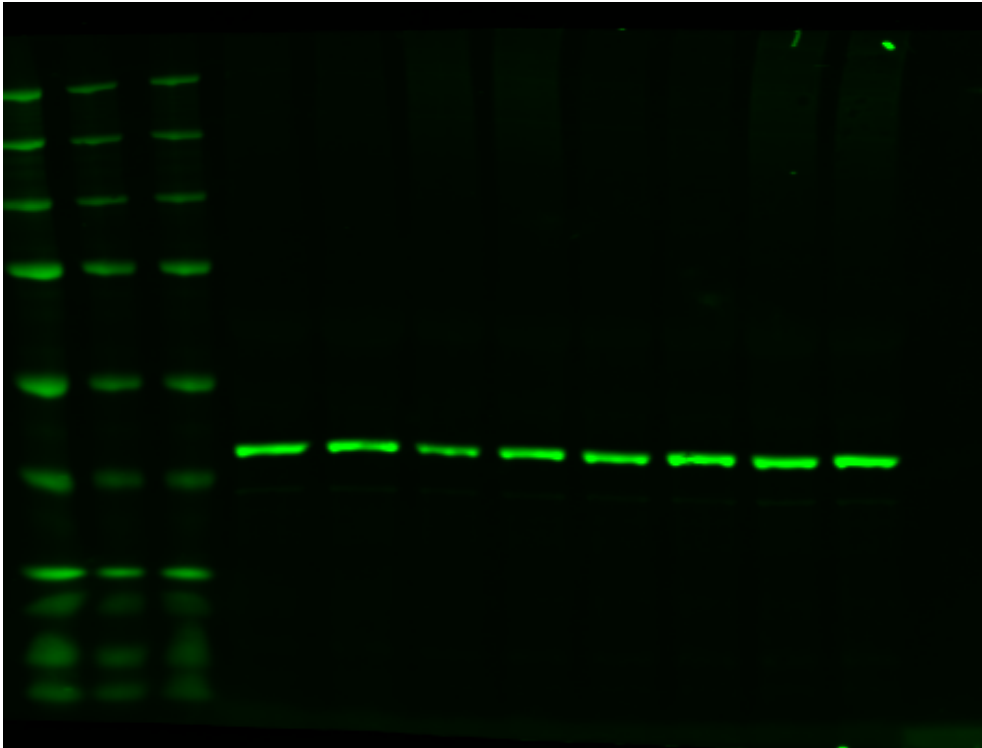

Supplement: Figure 3—source data 1. [file elife-52714-fig3-data1.pdf]
