# Supplementary material for: The switch-like expression of heme-regulated kinase 1 mediates neuronal proteostasis following proteasome inhibition
Source: eLife. 2020 Apr 24;9:e52714. doi: 10.7554/eLife.52714 (PMC7224698; doi:10.7554/eLife.52714)
Supplement: Figure 2—figure supplement 1—source data 2. [file elife-52714-fig2-figsupp1-data2.pptx]

## Slide 1
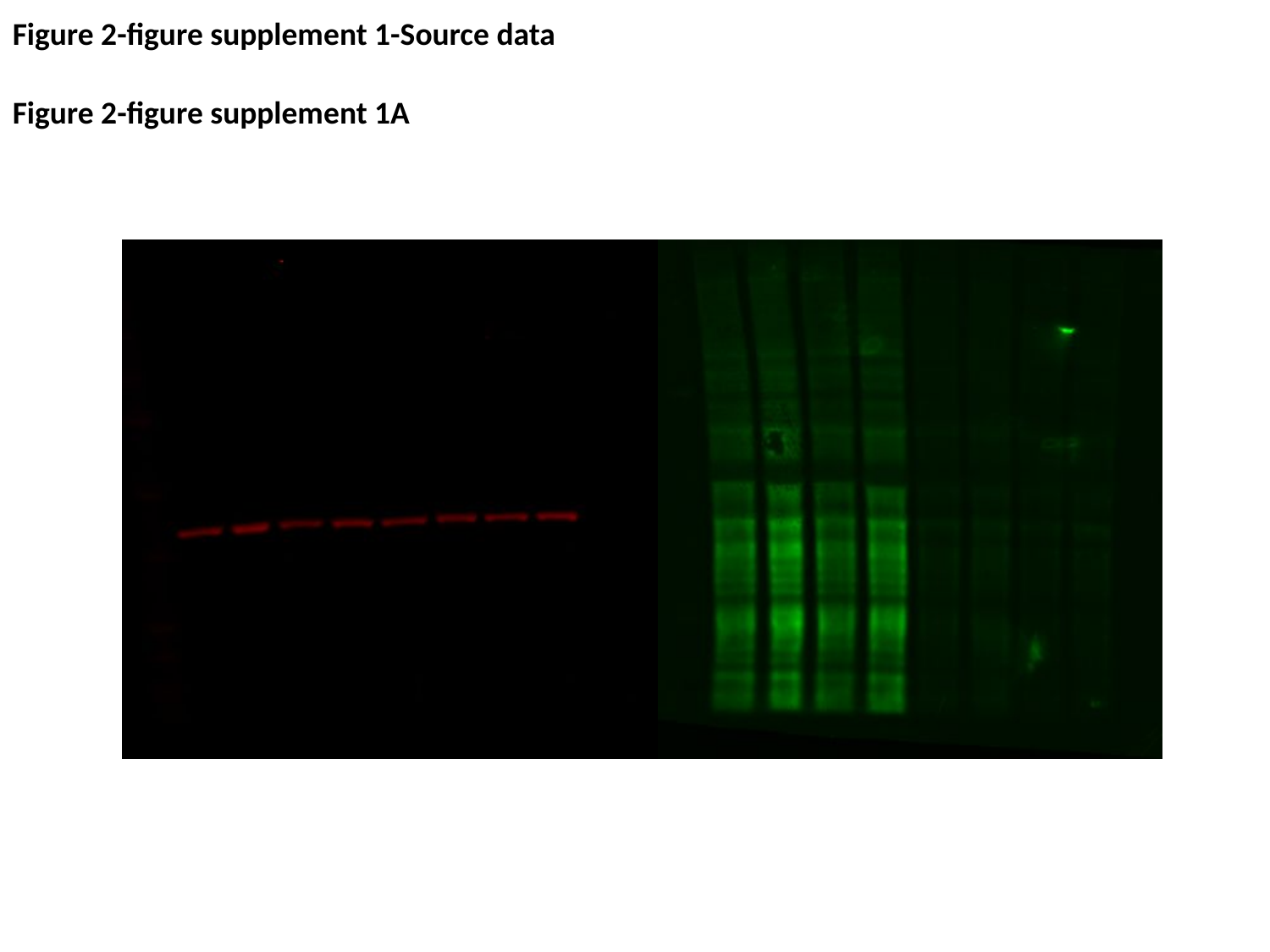

Figure 2-figure supplement 1-Source data
Figure 2-figure supplement 1A

## Slide 2
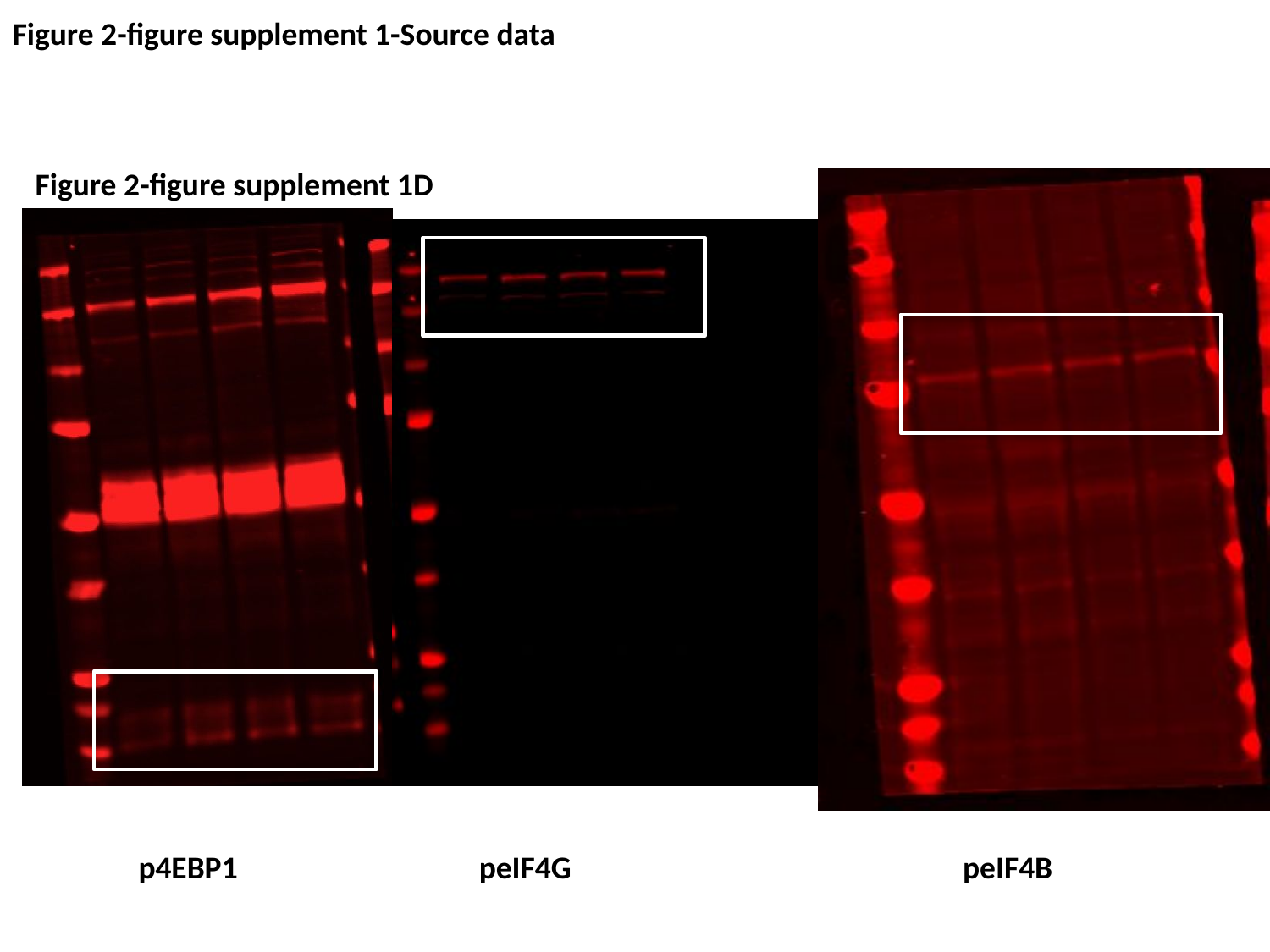

Figure 2-figure supplement 1-Source data
Figure 2-figure supplement 1D
p4EBP1
peIF4G
peIF4B

## Slide 3
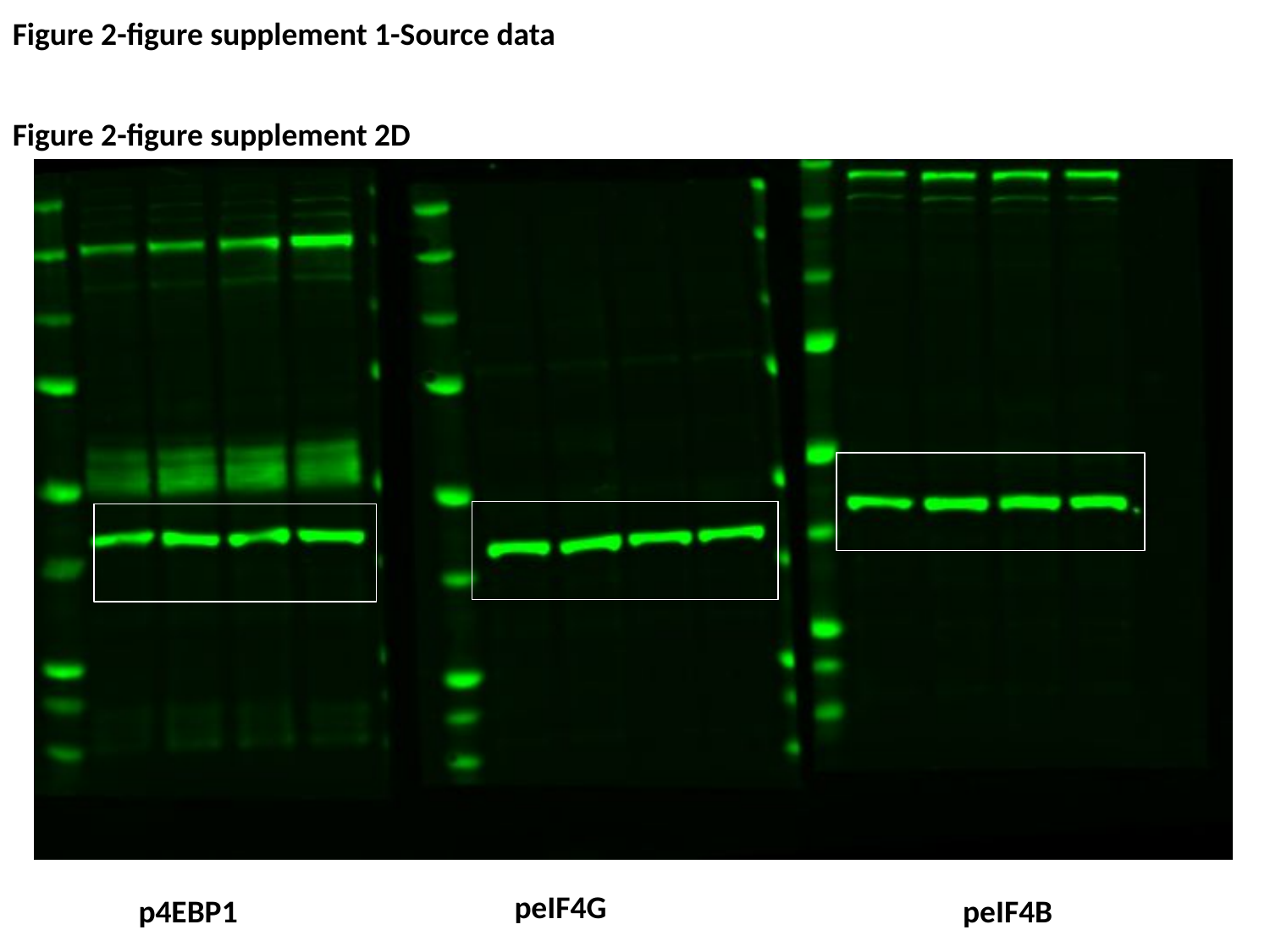

Figure 2-figure supplement 1-Source data
Figure 2-figure supplement 2D
peIF4G
p4EBP1
peIF4B
