# Supplementary material for: The switch-like expression of heme-regulated kinase 1 mediates neuronal proteostasis following proteasome inhibition
Source: eLife. 2020 Apr 24;9:e52714. doi: 10.7554/eLife.52714 (PMC7224698; doi:10.7554/eLife.52714)

Figure 3- figure supplement 1- source data

Figure 3-figure supplement 1C

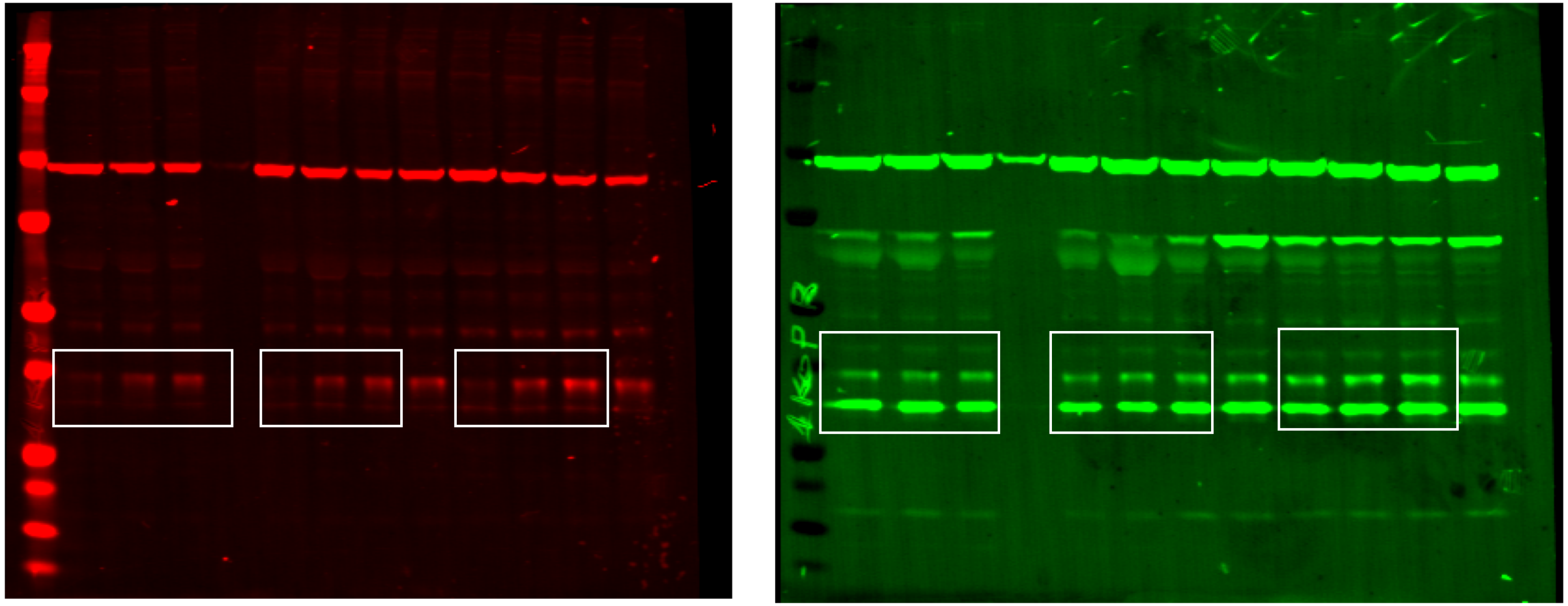

Figure 3- figure supplement 1- source data

Figure 3-figure supplement 1E

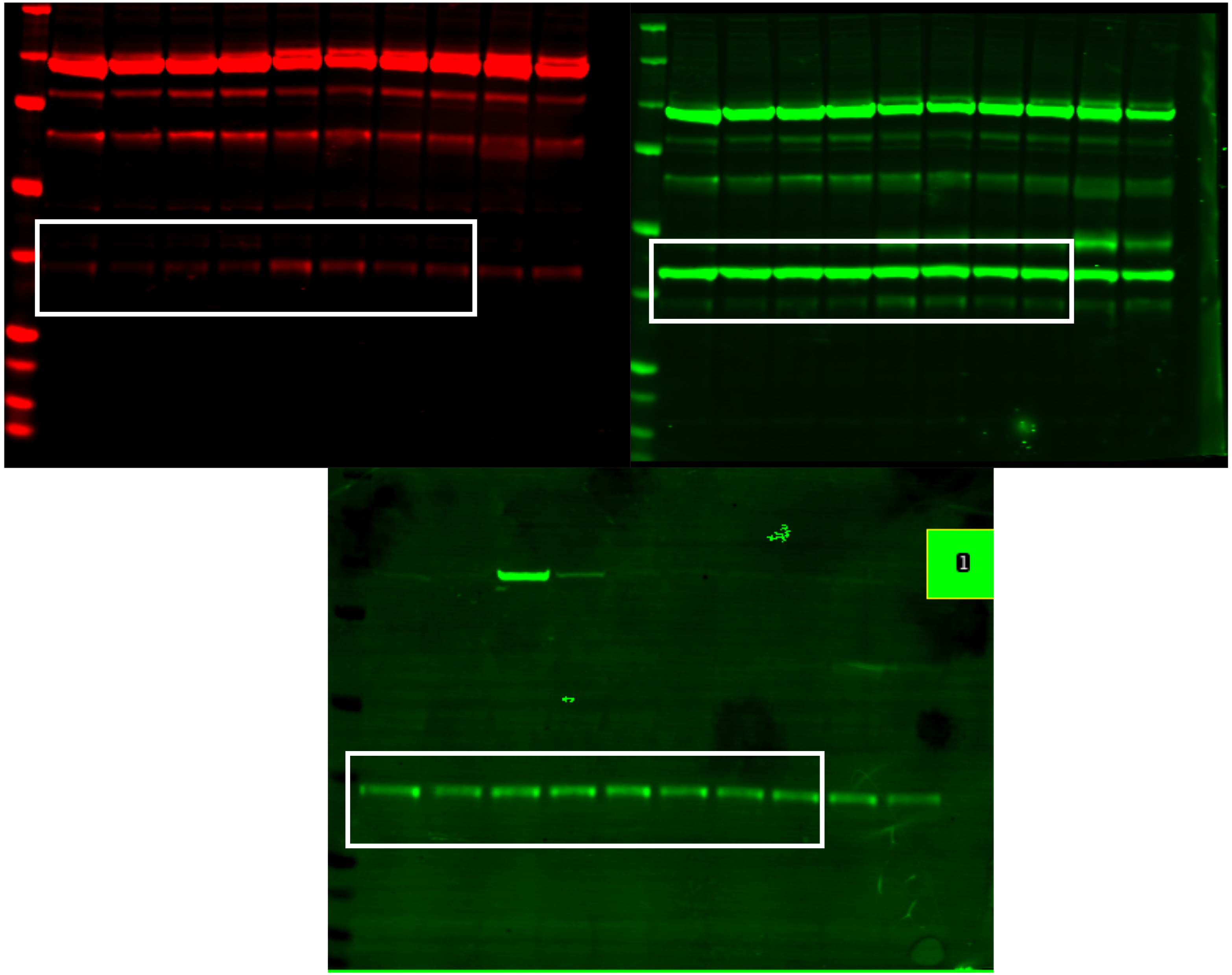

Supplement: Figure 3—figure supplement 1—source data 2. [file elife-52714-fig3-figsupp1-data2.pdf]
