# Supplementary material for: The switch-like expression of heme-regulated kinase 1 mediates neuronal proteostasis following proteasome inhibition
Source: eLife. 2020 Apr 24;9:e52714. doi: 10.7554/eLife.52714 (PMC7224698; doi:10.7554/eLife.52714)

Figure 4- figure supplement 1- source data

Figure 4- figure supplement 1A

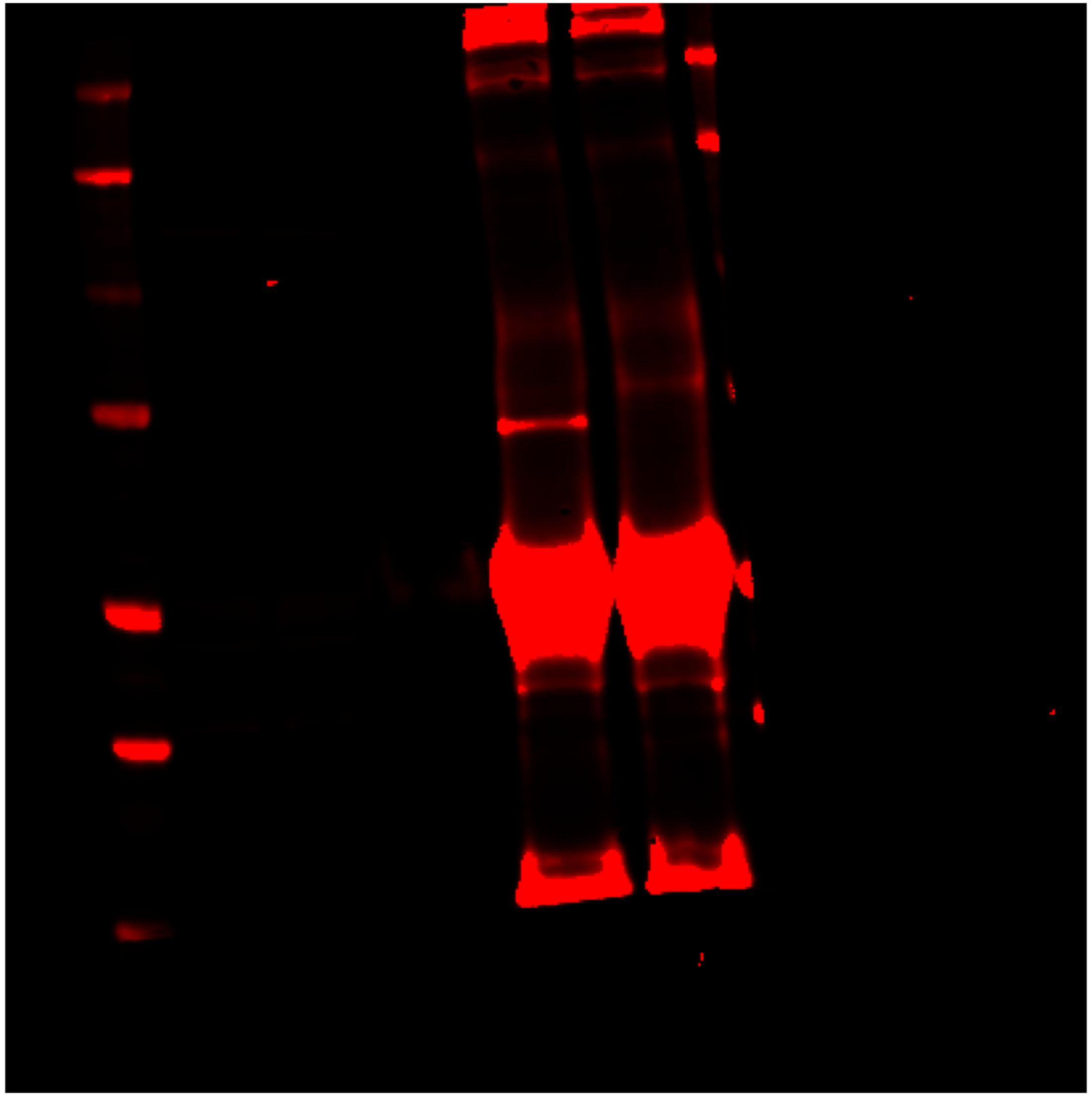

Figure 4- figure supplement 1- source data

Figure 4- figure supplement 1B

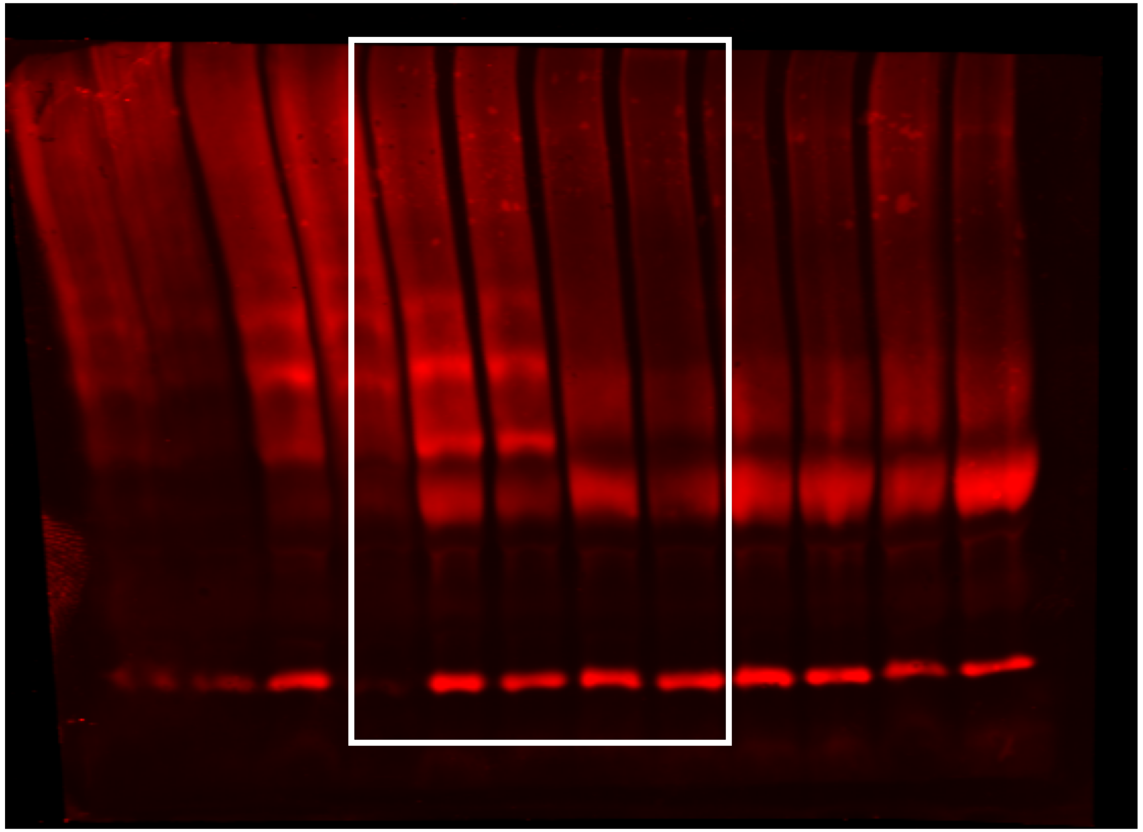

Figure 4- figure supplement 1- source data

Figure 4- figure supplement 1C

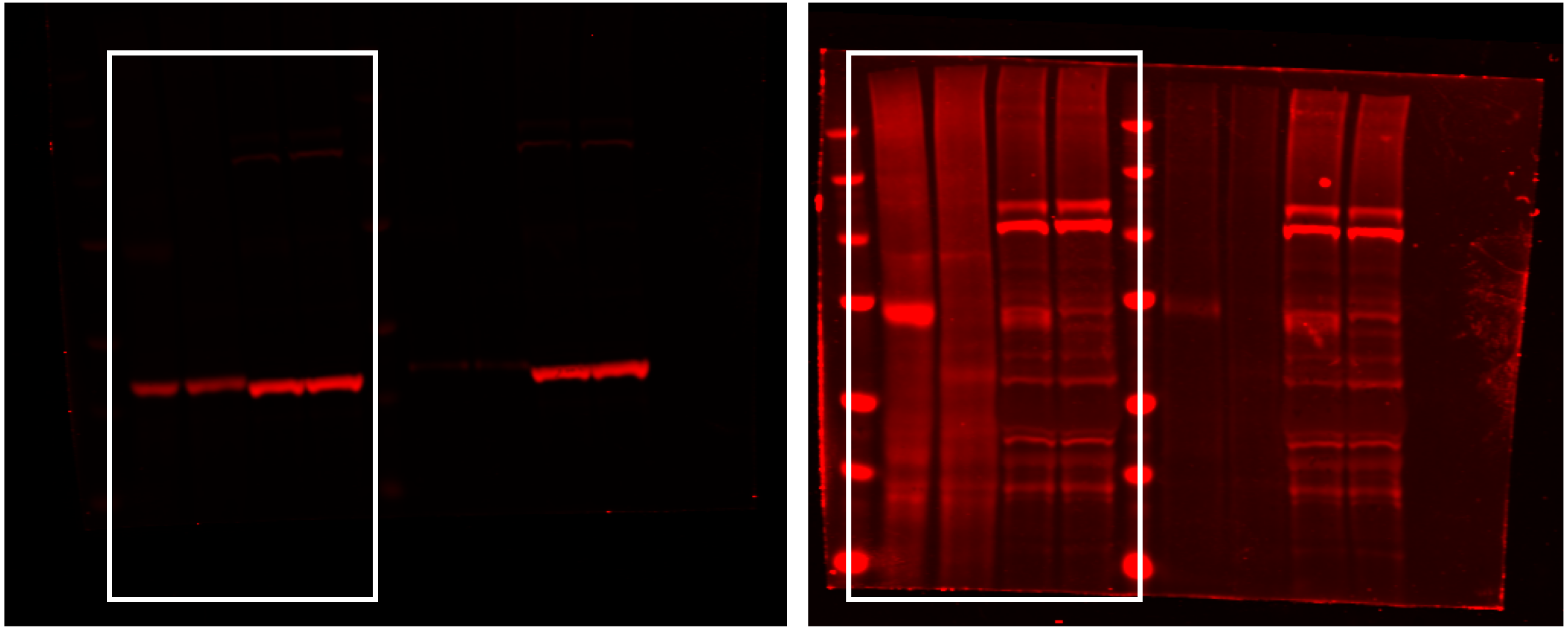

Figure 4- figure supplement 1- source data

Figure 4- figure supplement 1D

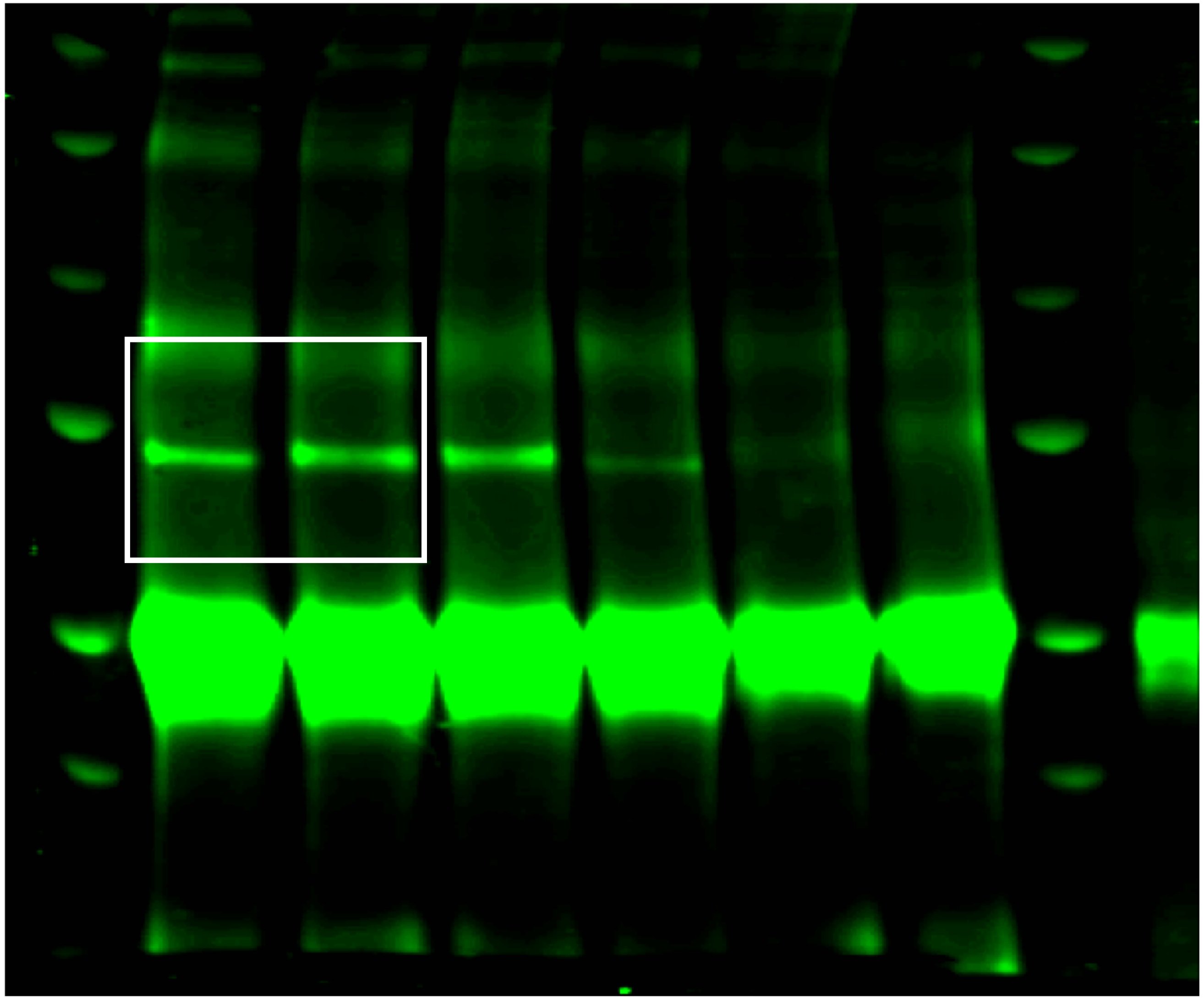

Figure 4- figure supplement 1- source data

Figure 4- figure supplement 1D and F

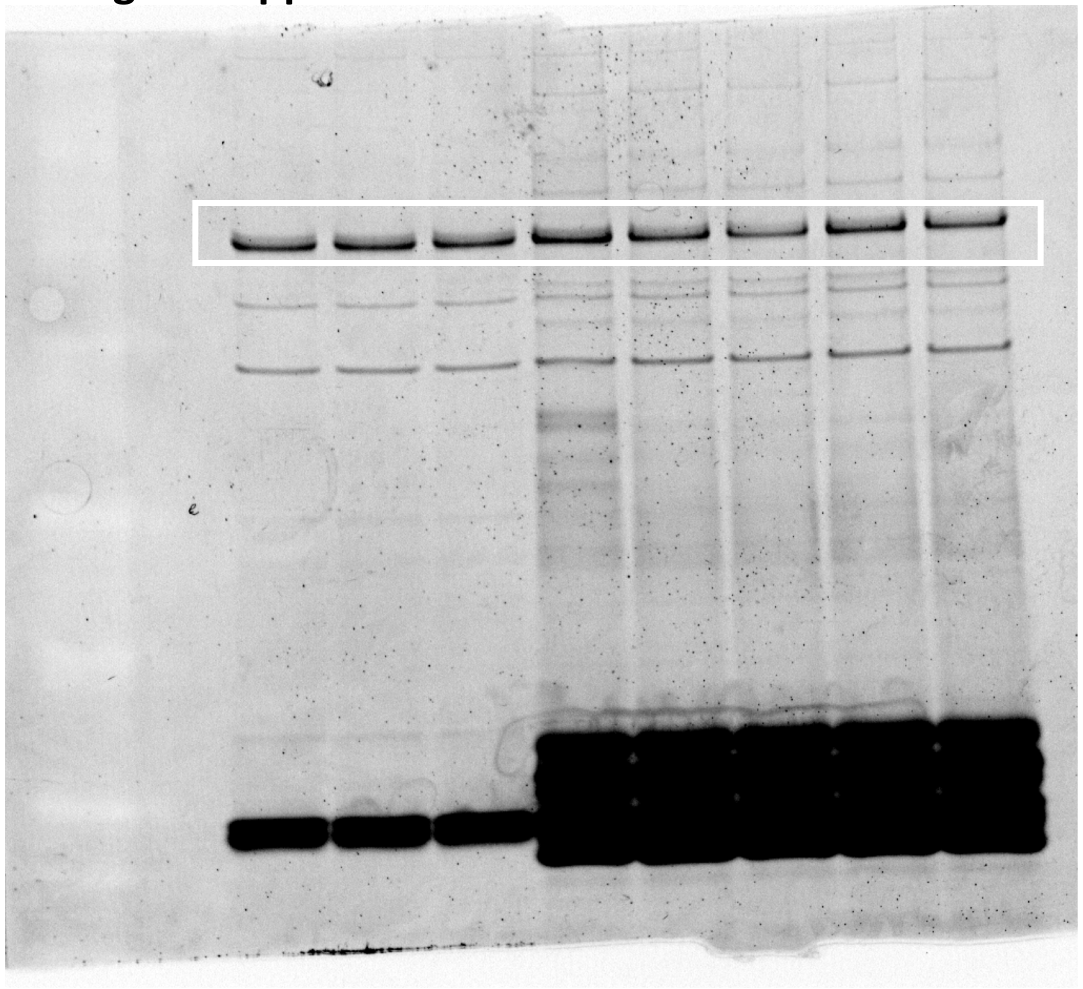

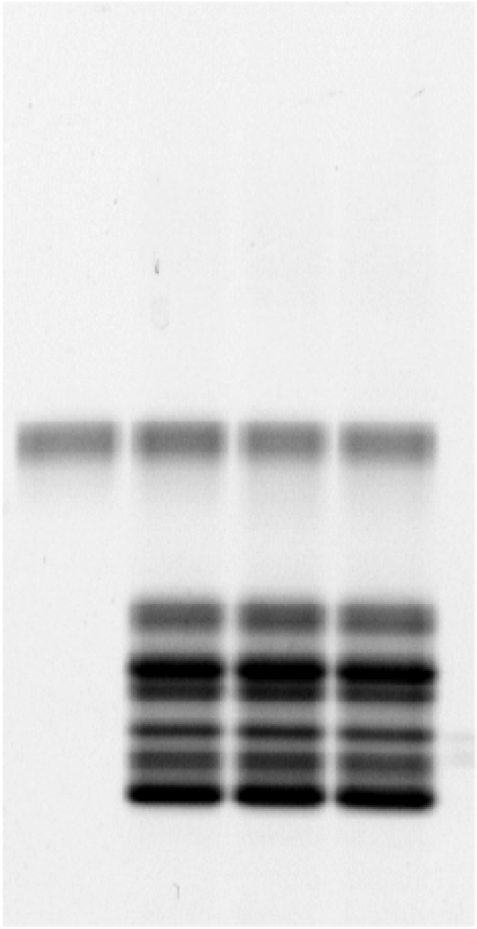

Supplement: Figure 4—figure supplement 1—source data 2. [file elife-52714-fig4-figsupp1-data2.pdf]
