# Supplementary material for: The switch-like expression of heme-regulated kinase 1 mediates neuronal proteostasis following proteasome inhibition
Source: eLife. 2020 Apr 24;9:e52714. doi: 10.7554/eLife.52714 (PMC7224698; doi:10.7554/eLife.52714)

## Figure 5- figure supplement 2- source data

## Figure 5-figure supplement 2I

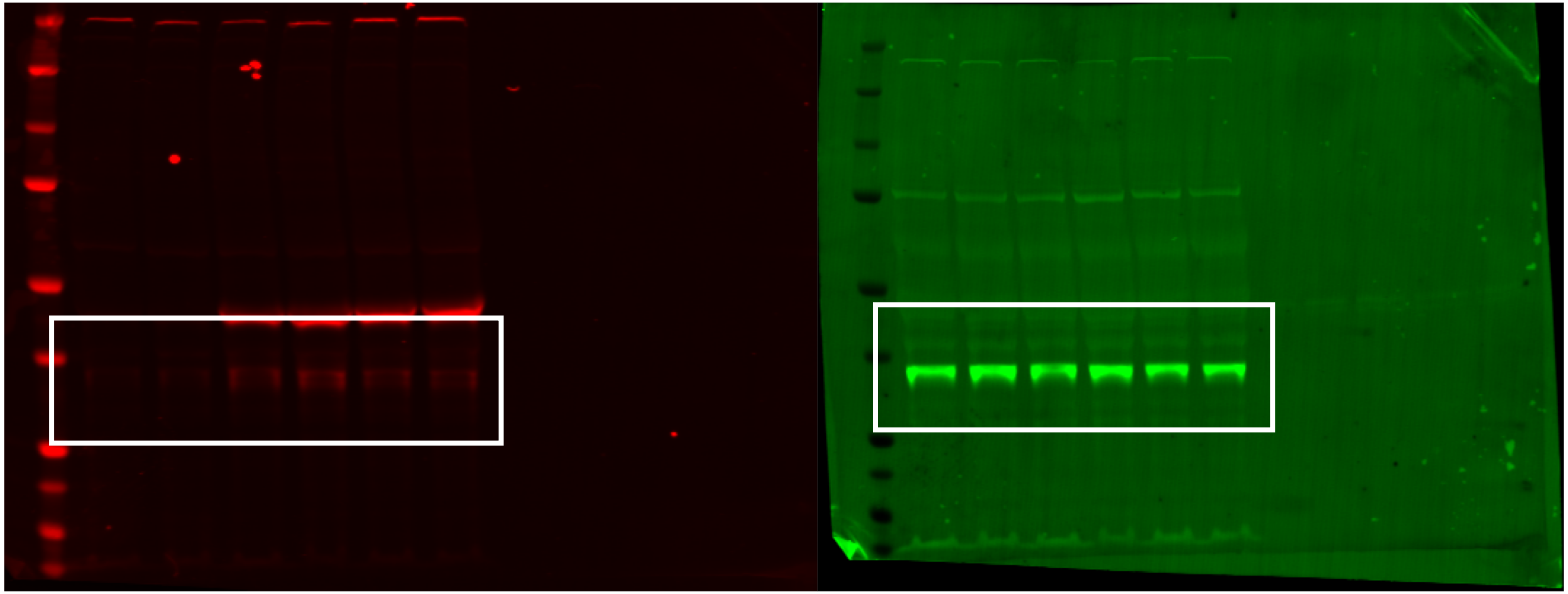

## Figure 5- figure supplement 2- source data

## Figure 5- figure supplement 2K

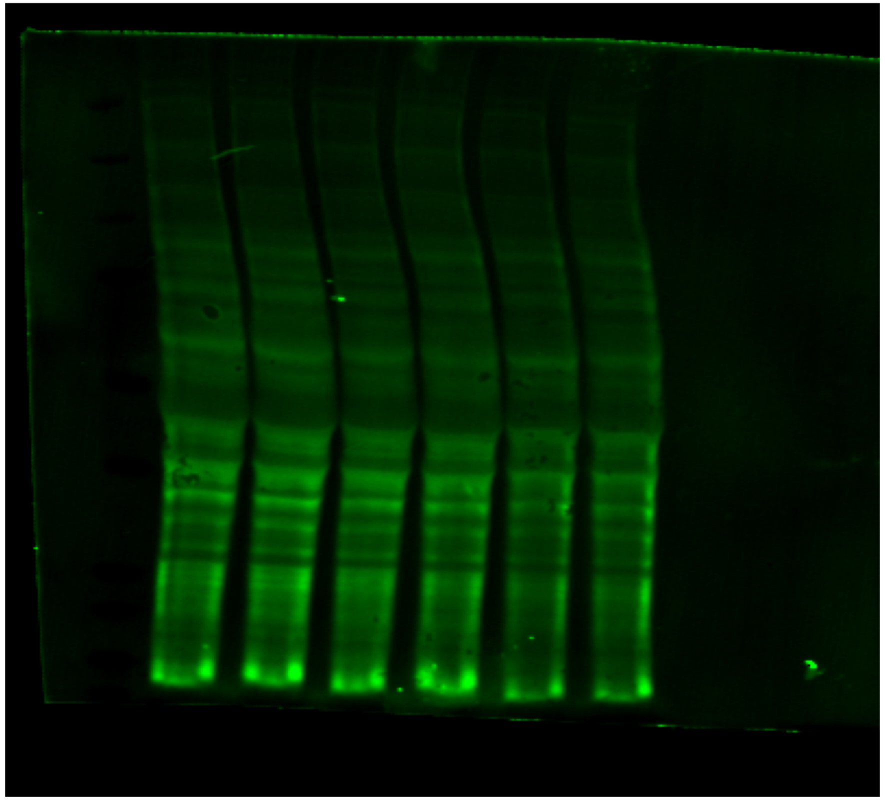

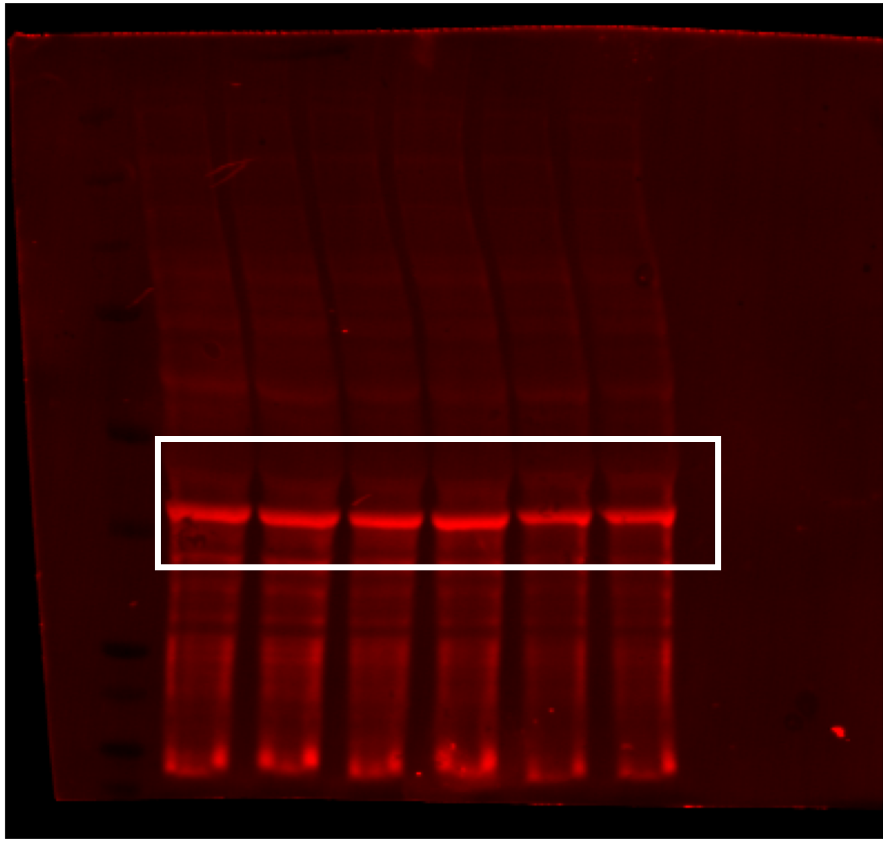

Supplement: Figure 5—figure supplement 2—source data 2. [file elife-52714-fig5-figsupp2-data2.pdf]
